# PROMISE: A Model of Insight and Equanimity as the Key Effects of Mindfulness Meditation

**DOI:** 10.3389/fpsyg.2019.02389

**Published:** 2019-10-22

**Authors:** Juliane Eberth, Peter Sedlmeier, Thomas Schäfer

**Affiliations:** ^1^Department of Psychology, Chemnitz University of Technology, Chemnitz, Germany; ^2^Department of Psychology, MSB Medical School Berlin, Berlin, Germany

**Keywords:** meditation, mechanisms, insight, equanimity, mindfulness

## Abstract

In a comprehensive meta-analysis on the effects of mindfulness meditation, Eberth and Sedlmeier (2012) identified a multitude of positive effects that covered a wide range of psychological variables, such as heightened mindfulness as measured through contemporary mindfulness scales, reduced negative emotions, increased positive emotions, changes in self-concept, enhanced attention, perception, and wellbeing, improved interpersonal abilities, and a reduction of negative personality traits. The present research aimed at developing and testing a comprehensive model explaining the wide range of mindfulness meditation effects and their temporal and causal relationships. In Study 1, interviews with meditators at different levels of experience were analyzed using a grounded theory procedure. The resulting model was triangulated and refined by concepts from both Western research and ancient Buddhist scriptures. The model developed highlights *equanimity* (reduction in emotional reactivity) and *insight* (alteration of cognitions) as the two key effects of mindfulness meditation that eventually lead to increased wellbeing. The model was pilot-tested with a large sample of meditators and non-meditators in Study 2. Data showed an acceptable fit with the model and indicated that meditators and non-meditators score significantly differently on the model’s core categories.

## Introduction

How does meditation work? Along with the growing interest in the effects of meditation techniques and the emerging evidence that these effects are astonishingly numerous (e.g., Eberth and Sedlmeier, 2012; Sedlmeier et al., 2012) particularly Western researchers have been theorizing about the potential mechanisms behind these effects (e.g., Shapiro et al., 2006; Dahl et al., 2015). While older approaches often referred mostly to the Western psychological concepts at their time (such as the idea that sitting in silence might lead to a *relaxation response*) newer approaches referred more closely to the ancient Buddhist thought system (such as the idea that trying to perceive oneself as separate from one’s thoughts or sensations during meditation leads to *non-attachment*). Although some mechanisms proposed are partially overlapping, and sometimes different terminology is used to refer to very similar ideas, there are a number of mechanisms that can be distinguished quite consistently from the literature (see Table 1). What all these mechanisms have in common, however, is that they have emerged from theorizing and quantitative measurement – thus disregarding what meditating individuals subjectively perceive as the predominant components of how meditation works within their experience. On these grounds, the aim of the present research was (1) to address the subjectively perceived mechanisms that lead to the positive effects of meditation, (2) to triangulate these perceptions with both the Western concepts of how meditation works and the mechanisms that can be extracted from the ancient Buddhist scriptures, and (3) to develop a comprehensive model that is informed by these three sources of information (Study 1), as well as to (4) to test this model empirically (Study 2).

**TABLE 1:** Proposed mechanisms in the Western literature of how meditation leads to its positive effects.

**Proposed mechanisms of meditation practice**	**References**
**Attention regulation** (training of either focusing attention to a specific perception, such as breathing, or widening it to all present stimuli)	Shapiro et al., 2006; Lutz et al., 2008; Hölzel et al., 2011
**Relaxation response** (deep relaxation through breathing exercise and repetition of a mantra)	Hoffman et al., 1982; Benson, 1993
**Interruption of mental proliferation** (active stopping of habitual lines of thought automatically triggered by specific stimuli)	Grabovac et al., 2011
**Metacognitive awareness and decentering** (effort to perceive one’s own thoughts, sensations, emotions etc. from a superior perspective and to realize that one is not identical to these phenomena)	Safran and Segal, 1990; Teasdale et al., 2002; Hayes et al., 2006; Shapiro et al., 2006; Brown et al., 2007; Schooler et al., 2011
**Changes in self-concept and self-perception** (reconsideration and change of personal beliefs, values, strengths etc.)	Hayes et al., 2006; Shapiro et al., 2006; Grabovac et al., 2011; Vago and Silbersweig, 2012
**Self-compassion** (training of a positive perception and behavior regarding the own person)	Baer, 2010
**Self-regulation** (ability to successfully “function” and to adapt to changes)	Shapiro et al., 2006
**Non-attachment/detachment** (state of freedom from any craving and aversion)	Shapiro et al., 2006; Brown et al., 2007; Grabovac et al., 2011; Vago and Silbersweig, 2012
**De-automatization/de-coupling** (reduction of abstract interpretations; concentration on pure sensation)	Deikman, 1966; Wenk-Sormaz, 2005; Kang et al., 2013
**Reduced bias in processing of self-relevant information** (and development of appropriate, not emotionally-driven behavior)	Vago and Silbersweig, 2012
**Exposition and relearning** (readiness to face unwanted stimuli or thoughts, resulting in reduced emotional activity toward these)	Baer, 2003; Brown et al., 2007; Hölzel et al., 2011; Vago and Silbersweig, 2012
**Positive reappraisal** (of stimuli that usually trigger anxiety and stress)	Garland et al., 2009
**Altered meta-emotions** (reduction of subordinate emotions, such as shame as a reaction to one’s anxiety)	Mitmannsgruber et al., 2009
**Enhanced body awareness** (heightened sensitivity toward bodily sensations)	Hölzel et al., 2011

## Study 1: Development of a Comprehensive Model of the Psychological Changes Induced by Mindfulness Meditation – a Qualitative Study

Since *mindfulness* meditation has caught the attention of researchers and the public much more than any other form of meditation technique (Grabovac et al., 2011; Hölzel et al., 2011; Eberth and Sedlmeier, 2012), the present paper is concerned with the psychological mechanisms that are responsible for the positive effects of this form of meditation. Following Kabat-Zinn (1990), Hölzel et al. (2011, p. 538) define mindfulness as “non-judgmental attention to experiences in the present moment” that “is typically cultivated in formal meditation practices, such as sitting meditation, walking meditation, or mindful movements.”

With our first study, we aimed to explore the effects of mindfulness meditation practices that meditators consciously noticed and attributed to their practice as well as the subjective explanatory models they had for these effects. In addition, we triangulated these qualitative data with both mechanisms that have been proposed in the Western literature and concepts from ancient Buddhist scriptures, resulting in the development of a comprehensive model of the process and mechanisms of meditation effects.

### Methods

#### Sample

We contacted several German meditation centers, gathered contact data from 35 practitioners willing to participate, and eventually interviewed 11 persons. Sampling was done according to a theoretical sampling procedure focused on maximum diversity in meditation experience, a balanced gender ratio, and the incorporation of representatives of the main Buddhist approaches to meditation in the West: Zen, Vipassana, and Tibetan. Data gathering was stopped when theoretical saturation was reached (the last interviews provided very little new information and were therefore used for model testing). Nine interviews were conducted in German, two were conducted partially or completely in English since the interviewees were not native German speakers. Three interviews were done by telephone and the other eight were face-to-face interviews. Table 2 summarizes the characteristics of the interviewees.

**TABLE 2:** Characteristics of interviewees, ordered by interview chronology.

**Sex**	**Age**	**Buddhist tradition**	**Meditation experience in years**	**Acts as meditation teacher**	**Number of retreats attended**
F	29	Tibetan	10	No	None
F	27	Kwan Um Zen	4	No	Some
M	54	Rinzai Zen	25	Zen	Many
F	35	MBSR	3.5	No	None
M	33	Vipassana (Goenka)	9	MBSR	Many
M	32	MBSR, Zen	1.5	No	None
M	59	Soto Zen	30	Zen	Many
F	42	Tibetan	7	Unclear	Unknown
F	38	Zen	4	No	Some
M	39	Vipassana (Goenka), MBSR	10	MBSR	Few
M	62	Theravada, Tibetan, Zen	23	Yes	Many

#### Data Collection

Data collection proceeded in three steps: First, we conducted five narrative interviews to identify consequences of the meditation practice that were considered as subjectively most relevant; second, we proceeded with three more focused interviews to explore the content and relationship between the identified concepts in more detail; third, in three further interviews, we returned to the narrative approach that was now enriched with a subsequent addressing of the initially identified core concepts to test the model. The narrative interviews started with some warm-up questions (e.g., when and why somebody started to meditate) and proceeded to the following prompt to tell a continuous story about their experiences with meditation (according to Willig and Stainton Rogers, 2008): “Can you remember the first time you noticed something had changed with you due to your meditation practice? If so, please tell me about that time.” If the interviewees stopped, the interviewer encouraged them to continue. The participants were instructed to stay close to their personal experiences rather than report abstract concepts they had learned from the literature or teachings on meditation. When the story was over, the interviewer asked participants to remember more occasions when they had noticed changes due to meditation. This process continued until the interviewee had the impression that all the important effects of meditation they were aware of had been discussed. The interviews were logged via voice recorder and transcribed with the F4 software (V3.1)^1^.

#### Data Analysis

The coding strategy was applied to every interview transcript successively. It comprised five steps: (1) identification of relevant passages in the text that referred to changes noticeable in daily life, changes in consciousness during meditation practice and how meditation may be used instrumentally, (2) open coding according to grounded theory (Glaser and Strauss, 1967) that yielded semantic primary codes (see also Braun and Clarke, 2006), (3) summarizing the primary codes in broad descriptive categories – resulting in a preliminary coding scheme, (4) condensing the primary codes to abstract secondary codes within the descriptive categories, (5) restructuring the secondary codes across the descriptive categories – resulting in a refined structured coding scheme, (6) refining the categorical system according to the secondary codes with the result that it advanced from a descriptive reservoir to theoretical concepts. Additionally, starting from Interview 8, we constructed and adapted a graphical model representing all categories identified so far, the secondary concepts, and their connections among each other. Eventually, all secondary codes in the final model were again cross-checked for homogeneity by going back to the corresponding interview passages. Coding was primarily done by the first author, using the MAXQDA software (Kuckartz, 2001), and regularly discussed with the second author; construction and adaptation of the model was a joint process by all three authors. Any inconsistencies or difficulties while coding were discussed at any time, although this was necessary very seldom. Coding consistency was not explicitly examined.

#### Triangulation With Western Concepts and Buddhist Scriptures

As mentioned above, there have been a lot of concepts developed in Western research on how meditation might work, which we have summarized in Table 1 (see also Hölzel et al., 2011; Gu et al., 2015). Notably, there is not always consensus on how to clearly differentiate between the components of mindfulness practice (what people do when meditating), the mechanisms the meditation practice initiates, and the eventual effects these mechanisms result in. For instance, *acceptance* (of positive as well as negative thoughts, emotions, sensations; sometimes also referred to as *cognitive flexibility*) might be conceptualized as a mechanism; but in the literature, it has most often been conceptualized as something that is actively practiced during meditation, thus being a component of practice (see Hayes et al., 2004). Anyway, we took such concepts into account when coding the interview data, although they are not listed as unique mechanisms in Table 1.

While the construction of the model of meditative change (see below) was primarily based on the qualitative data, the mechanisms listed in Table 1 were used to refine the model and augment it with a more detailed understanding of the possible processes. This strategy was used because some processes might be unlikely to be accessible to conscious verbalization in the course of interviews. Nonetheless, most of the mechanisms were occasionally mentioned – directly or indirectly – by the interviewees. Mechanisms not mentioned directly in the interviews, were tested for plausibility and compatibility with the model and eventually incorporated if appropriate.

At the same time, the interviews identified some important concepts that had not been defined and discussed in the Western literature so far. Therefore, we turned to ancient Buddhist scriptures to further triangulate our model. We focused on interpretations of the Nikayas – a collection of scriptures that is fundamental to the Theravada Buddhism, a tradition that is considered to have very old roots, and that is also accepted by most of the more recent Buddhist currents. Within the Nikayas, we identified several texts (translated by Bodhi, 2005; Nyanaponika, 2006; Walshe, 2007; Kornfield, 2008; Thanissaro Bikkhu, 2013) that provided detailed information about two major categories identified in the interviews (*meditative insight*: e.g., AN2:iii, 10;I61, Sammaditthi Sutta I46-55, MN109, SN22.7;III15, SN22.45, SN28:1-9;III235-38, AN5.28, AN8.2, AN10.13, and *equanimity*: e.g., AN8.6, SN46:55, SN14.1;II.140, AN10.13).

After integrating the information from the three sources (interviews, Western concepts, Buddhist scriptures), we worked up a model on the process and mechanisms that lead to the positive effects of mindfulness meditation. We dubbed it the *PROMISE* model (“the process of mindfulness meditation leading to insight and equanimity”).

### Results

#### The General Process of Meditative Change

**FIGURE 1:**
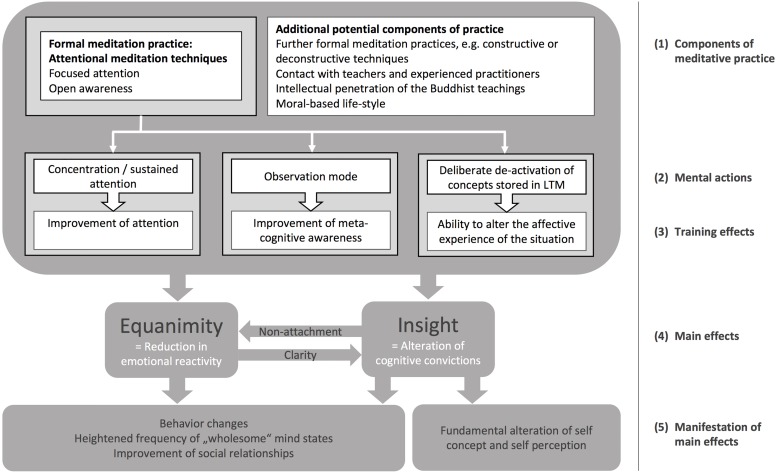
The general process of meditative change.

The *PROMISE* model represents a causal/temporal sequence. Specifically, it is partitioned in five steps (see Figure 1): (1) the components of the meditation practice, (2) the mental actions during mindfulness meditation, (3) abilities that are trained by conducting these mental actions repeatedly, (4) profound changes in emotions and convictions, (5) important effects in daily life.

##### Step 1: The components of the meditation practice

This step covers the building blocks of a meditation practice. First of all, it contains the meditation technique itself. Meditators conduct many different meditation techniques, often varying both within and between different sessions (see also Lutz et al., 2008). There are some approaches to distinguishing different meditation techniques (see Matko and Sedlmeier, 2019). For example, Dahl et al. (2015) established three categories: attentional meditation (targeting meta-awareness and experiential fusion), constructive meditation (targeting reappraisal, perspective-taking, and self-schema), and deconstructive meditation (targeting self-inquiry and insight). The *PROMISE* model applies exclusively to attentional meditation though our interview partners occasionally reported also using constructive or deconstructive techniques (primarily loving-kindness meditation and Vipassana or analytical meditation, respectively). However, the qualitative analysis concentrated on attentional techniques since these were far more common and homogenous. Within the attentional meditation family, we differentiate between focused attention mediation, that means “sustaining selective attention moment by moment on a chosen object” (Lutz et al., 2008, p. 6) and open monitoring meditation, that means remaining “only in the monitoring state, attentive moment by moment to anything that occurs in experience without focusing on any explicit object” (Lutz et al., 2008, p. 6) Yet, the bare meditation technique is not the only thing that sets meditators apart from non-meditators. Rather, practitioners also consult meditation teachers personally or through different media like books or videos; a lot of practitioners read ancient scriptures or interpretations thereof and with that try to understand the Buddhist ideas; and many meditators try to implement the Buddhist thought in their daily life and, consequently, try to follow at least some of the ethical guidelines (e.g., not killing any life with the own behavior or avoid false and harsh speech). These three components – learning with a teacher, reading Buddhist literature, and heeding ethical principles – can be considered experiences that may accompany the actual practice of meditation and can generate or amplify positive effects. In the current version of the PROMISE model, the effects of the accompanying factors are not yet elaborated (see Discussion).

##### Step 2: The mental actions during mindfulness meditation

Let us now focus on the meditation techniques itself. As stated above, the PROMISE model applies to focused attention meditation and open monitoring meditation techniques. These techniques are characterized by three mental actions to differing degrees: selective attention and concentration, monitoring of mental experience, deactivation of certain cognitions. Focused attention meditation is characterized by a very high degree of selective attention or concentration. In our interviews the practitioners described it as “one-pointedness of mind” or that the “mind is sharp like a pencil.” In contrast, open monitoring meditation is not characterized by selective concentration but by monitoring one’s own mental experience. This state of mind was labeled “observation mode” by some of our interview partners. While being in that mode, inner states (including thoughts, emotions or impulses, but also perceptions obtained through the sense organs) are perceived in their sequential and parallel manifestation. One could also say that the observation mode means to constantly bring all perceivable aspects of the very present moment into consciousness. Equally important for focused attention and open monitoring meditation techniques is a further mental action that supports the focusing and monitoring, respectively: the active letting-go or deactivation of expectations, judgments and current thoughts, opinions, ideas, and other cognitive products. In focused attention meditation techniques, this active release is needed when thoughts distract the practitioner from their meditation object and it allows for reorientation. The same applies to open monitoring meditation techniques, where practitioners might further elaborate the observed mental objects. When such an elaboration is identified, dropping the cognitive processes or their causes permits the practitioner to return to their monitoring stance.

##### Step 3: Abilities that are trained by conducting these mental actions repeatedly

Usually, meditation is practiced on a quite regular basis: mostly once or twice a day, at least once or several times a week. This repeated conduct of the three main mental actions identified in Step 2 leads to an improvement in the abilities that are needed to perform these actions. First, to focus attention (FA) on a meditation object, the practitioner requires the ability of selective and executive attention. Respective neural networks are expected to be strengthened by regular FA practice (Lutz et al., 2008; Hölzel et al., 2011). Second, to observe mental events, the practitioner has to step out of their experience, a phenomenon that is well known in contemporary literature as metacognitive awareness or decentering (Safran and Segal, 1990; Teasdale et al., 2002). Third, by adopting an attitude of openness, acceptance and non-attachment to present experiences on a regular basis, the practitioner gains the ability to alter the affective quality of any experience to be more neutral.

##### Step 4: Emotional improvements and alterations of convictions

The improvement of abilities that are used during the meditation sessions is the first long-term effect one can expect after starting to meditate on a regular basis. But the effects of meditation are more far-reaching. The improvement of the trained abilities, the deliberate use of meditative mental actions in daily life, and the just mentioned aspects that often accompany formal meditation practice trigger mechanisms that bring forth two very important effects of meditation: equanimity and insight. The PROMISE model exposes these two as *intended* effects for two reasons: first, almost all mechanisms of action described by our practitioners lead to equanimity and/or insight and, second, Buddhist scriptures identify equanimity and insight as the crucial acquirements to overcome two basic hindrances for enlightenment: “When serenity is developed … all lust is abandoned” and “When insight is developed … all ignorance is abandoned” (AN 2: iii, 10; I 61; translated by Bodhi, 2005). Notably, only one model published in the West focuses on these two components (Grabovac et al., 2011). Other models barely mention them, if at all. In the following, equanimity and insight will be described in detail.

On the basis of the interview statements, equanimity is defined as reduced frequency and duration of emotional reactions, such as boredom, self-blame, anxiety, guilt, greed, envy, and many more. However, emotional activity does not completely cease. A persistent absence of any emotional activity would rather represent indifference, which is seen as a “near enemy” of equanimity. Instead, the old Buddhist scriptures clearly define specific classes of situations in which emotional reactions should attenuate. These are situations that evoke emotions due to the presence of gain or loss, honor or dishonor, praise or blame, or pleasure or pain (AN8.6; translated by Bodhi, 2005). The reason for a reduced emotional activity in such situations is said to be a weakening in the desire for pleasurable sensual experiences as well as in the resistance against unpleasant experiences. These desires and resistances can be caused by the ignorance or unknowing of the impermanence (all phenomena constantly change and cease eventually) and the un-satisfactoriness of all phenomena (since all phenomena are ephemeral, nothing in the material world can bring about permanent happiness). Since individuals strive for things that cannot bring permanent happiness (as material things, work-related advancement or gaining admiration by others), they will suffer when these things remain absent or disappear. By contrast, an experienced meditator would recognize the emotion-evoking feature of the situation (e.g., praise or blame) but not experience a desire or resistance that would prolong or intensify the emotion. Instead, he would be able to detach from that pleasurable or unpleasant situation rapidly and go on to the next present-moment-experience. Said differently, the lower frequency, duration, and intensity of the experienced emotions should not be confused with a less frequent or shallower experience of emotion-evoking stimuli. What changes is only the extent to that they are conceived as pleasurable or unpleasant (see also Perlman et al., 2010).

The second category of intended meditation effects is the attainment of insights. As insights, we define convictional alterations that are accompanied by a subjective feeling of deep understanding and by changes in perception, judgment and/or behavior. The content of insights was very diverse both within and between our interviewees. According to Buddhist scriptures, they contain the discrimination between wholesome and unwholesome phenomena, insight into the fundamental characteristics of all being (namely impermanence, conditionality and unsatisfactoriness), insight into the existence of suffering as well as its origination and cessation, and insight into the nature of the self, resulting in a de-identification with the own body, feelings, perceptions, and consciousness. Among the reports of our interviews, we could distinguish between two general categories of insights: (1) some were cognitively well represented (like the realization of necessities and needs, changing perspectives or the recognizing that own opinions, beliefs and ideas have been wrong) and in most instances, they enabled insights into the functioning of the mind; (2) others were experience-based, intuitive, and not yet verbally represented. Accordingly, for our interviewees, the second insight category was difficult to put into words. The content touched mainly fundamental changes in the perception of the self and world.

##### Step 5: Endpoints relevant for the practitioners

The emotional improvements and convictional alterations manifest in various changes noticeable to the practitioner. First, they lead to an increasing frequency of wholesome mind states like feelings of trust, connectedness, and compassion with oneself and with others, strengths, contentment, or well-being. Next, they lead to an altered behavior in that it becomes more conscious and consequent. The conscious conduct of behavior is characterized by a high level of attention concentrated on stimuli connected with the current task and it prohibits doing more than one single activity at once. A consequent conduct of behavior is characterized by a shorter temporal delay between the recognition of the necessity of an action and its actual realization. The reason for that shortened lag might on the one hand be found in a decreased tendency to ruminate and on the other hand in a decreased tendency for egocentric influences informing the decision to get an action started. Last, it leads to an improvement of interpersonal relationships in that these become more straightforward and uncomplicated. The meditators reported that when egocentric tendencies are weakened, they were able to give the other person more space, which is very much appreciated by that other person. On the other hand, empathy is strengthened by practicing acceptance, equanimity, and self-insight, allowing the practitioner to infer more reliably what is going on in the other person.

The general process of meditative change is thought to organize the broad range of observed meditation effects in terms of chronology and causation. Now, after having identified what the steps of meditative change are, it is of major significance to explain *how* meditation leads to these effects. Thus, in the next paragraphs, we examine the mechanisms derived from the three sources that connect the steps of meditative change.

#### The Mechanisms Leading to the Positive Effects of Mindfulness Meditation

##### Training effects

The first mechanism was already explained in Step 3. All abilities that are used in establishing the meditative state are trained because they are utilized on a regularly basis. More specifically, the meditators train their ability to direct their attention to sensations they want to perceive, to detach their attention from sensations they do not want to perceive anymore (e.g., conceptual thoughts), and to monitor the own experience including the ability to partly step out of the stream of experience.

##### Role models

Role models are especially important for the development of equanimity and non-attachment. Role models might be meditation teachers and monks from Eastern or Western centers, but also companions in meditation groups and retreats. Practitioners might attend teachings or read ancient or contemporary books and learn how having internalized the Buddhist teachings manifests in observable behavior, in exemplary mental inferences and in emotional reactions. In this way, on the one hand, they reach a deeper understanding of the meaning of equanimity and, on the other hand, they learn observable behaviors and reactions representing calmness that foster internal calm.

##### New information

In the course of meditative practice, persons can get novel information by various routes: (1) reading ancient Buddhist texts, their interpretations or contemporary Buddhist teachings to penetrate the Buddhist thought system, (2) intentionally deactivating self-relevant concepts stored in long-term memory, which allows additional information to be processed, (3) taking up an observing stance toward internal experiences and, as result, getting to know themselves better, (4) using particular insight meditation techniques to deconstruct the experience and learning about the way both they themselves and the social world around them operate. In the following, the mechanisms will be described in more detail.

First, reading Buddhist literature and the personal contact to experienced meditators can offer inspiration for new perspectives and hence for the attainment of insights. Normally, it is assumed that a person has to practice formal meditation at least additionally to experience the theoretical knowledge, but there are also reports about students of the historic Buddha who reached enlightenment by merely listening to the teachings (Nyanaponika and Hecker, 2003). Apart from such exceptions, understanding the teachings might operate upon assimilation and accommodation processes (see Siegler et al., 2011). That is, the practitioners try to integrate the information in their cognitive system. If this is not possible, they might try to validate it in the course of formal meditation or informal mindfulness practice. When finding supporting evidence, they have to restructure their cognitive system so that it is able to differentiate between the old and the new information. To give an example: Say, a student learns that there is no stable self. However, his self-perception immediately suggests a stable self. Then, he does formal meditation, gains new experiences and, consequently, comes to the result that there is the experience of a stable self in daily life that is actually an illusion or psychic construction. Furthermore, teachers can give guidance and recommend certain meditation methods or everyday practices that foster certain insights one is able to attain. And, lastly, Buddhist literature and teachers can help practitioners bring novel or mysterious experiences in line with meaningful explanations.

The next mechanism is triggered by the practice of letting go, or the intentional deactivation of self-relevant concepts stored in long-term memory. Those concepts include, for instance, expectations, convictions, habitual judgments, and they guide attention and information processing. When those concepts are deactivated, experiences are made that normally would have been biased by attentional processes, like hypervigilance, disengagement delays, or avoidance. These altered experiences may act as a corrective and may extinguish the former incorrect conviction. A similar process has been described by Vago and Silbersweig (2012) and Levin et al. (2015) who termed it decoupling.

Most Buddhist meditation techniques entail an observing stance toward internal experiences as sensorial and bodily experiences, thoughts, motivations, emotions, moods, and more subtle internal states. This observation practice should be as non-judging and non-identifying as possible, which allows for letting the observed stimulus go after perceiving it and prevents a subsequent mental activity triggered by that stimulus. In everyday consciousness, such stimuli often trigger mental events like inner stories, conversations, and movies that, consequently, occupy processing capacity. As a result, this capacity is not available for processing the information of the present moment: sensorial and bodily experiences as well as first-order mental events (emotional reactions or mental responses like the activation of automatic thoughts patterns, expectations, needs and own limitations or impulses for acting). Again, first-order mental events refer directly to a stimulus of the present moment and differentiate from later (higher-order) mental events that refer to abstract mental operations that are principally independent in time – you could postpone thinking about an exam that will take place next week, but you cannot postpone your wish to run away when you get a difficult question in an oral exam. With the non-judging and non-identifying observation of information, the present moment unfolds, the meditator gets to know himself very well and becomes more and more able to distinguish between incoming stimuli and added subjective reactions. He might gain important insights like: “it’s me that makes suffering, it is not the situation itself.” Just observing, not reacting, allows the practitioner to experience the impermanence of everything observed and also the situational specificity of mental contents. This results in an even deeper non-identification with own mental contents and insights like: “it is just thoughts, it is not reality, and it only becomes my reality if I believe the thoughts represent reality.” The observation mode as it is described here is not just part of formal meditation practices. If it is transferred to everyday activity, it can be particularly effective since it is practiced for a longer period of time and since it is possible to register genuine reactions to manifold stimuli in real-time.

Due to the observation mode, practitioners learn about their own mental landscape and, therefore, gain information that was already there but had eventually to be attended. In formal mediation sessions, practitioners can also gain information that is completely new for them. The interviewees reported altered states of consciousness during deep meditative states: dissolution of the perception of the body, self, space, and time, states without thoughts, states of sensual openness (in which the normal constraints of the sense organs vanished), a deep awareness of single elements of consciousness, and experiences of unity, connectedness, pleasantness, and sometimes extreme happiness. Often, practitioners reported attentional alterations connected with these states. Attention was described as open, panoramic, automatic, easy and effortless. A characteristic feature of this altered attention is that stimuli from different sense organs and from different directions are processed in parallel and simultaneously. Note that in early Buddhism, parallel processing does not exist. Perhaps our interview partners tried to point to the fact that the stimuli are present in the external world and are processed in a rapid sequence that subjectively occurs as being parallel (see also Sedlmeier, 2016; Sedlmeier and Srinivas, 2019). All the described experiences represent by then unknown states of consciousness for a practitioner that seem to be hardly explainable to someone who has not yet experienced such a state. They seem to trigger insights that are also not easy to verbalize. Interviewees used more abstract expressions and sometimes metaphors known from Buddhist literature to refer to these insights and, upon request, to explain these, they had to take an inward-turn for finding suitable words and examples. Examples of such insights include: “we believe our mind has certain boundaries that actually do not exist” or “it is an illusion that something does exist.”

From these meditation experiences, states without thought activity are of particular interest because they seem to be an important facilitating factor for the realization of insights. Our interviewees termed this state “clarity.” We defined clarity as a state of consciousness that is characterized by the reduction or complete termination of all automatic conceptual thinking in favor of an improved perception of sensual and mental stimuli. In consequence, this state of consciousness gives space for intuition and pre-conscious content to become fully aware. All thought activity is released and ascending impulses and thoughts are observed. Practitioners reported that they gained an intuitive understanding for the becoming, maintenance, and ceasing of situations and circumstances. They attained insights resulting from a change in perspective, from detecting connections between things, or from recognizing necessities. In some cases, they were able to solve concrete problems or found answers to the “great questions of life.” Notably, it often appeared that questions and problems practitioners sought to solve during meditation, suddenly disappeared because the practitioners realized that these had just been based on erroneous presuppositions.

##### Emotion regulation

There are a lot of mechanisms that can be subsumed under the concept of emotion regulation.

First, interviewees reported intense experiences during meditation, especially characterized by extreme anxiety. During meditation, they did not avoid or suppress these emotions but went toe-to-toe with them, resulting in a by then unknown intensity of the respective emotion. Partly guided by experienced meditation teachers, they went through the emotion and, consequently, experienced their impermanence. By means of such experiences, they gained confidence in the impermanence of emotions so that, hereafter, in daily life it became easier for them to stand emotions without fighting them back. Additionally, the immense intensity of the emotions during meditation put daily emotions and sentiments into perspective so that these could be tolerated more easily. These descriptions of experience parallel the well-known psycho-therapeutic mechanism of exposure and relearning (see also Hölzel et al., 2011). Extremely intense emotions occur in at least one third of all meditators (estimated from our interview results and the results of Lomas et al., 2015) at least once within their meditation career.

A second emotion-regulation mechanism that has been described by our interviewees parallels the idea of inhibiting the process of mental proliferation (as proposed by Grabovac et al., 2011). In short, mental proliferation means that the appraisal of a sensation as pleasant or unpleasant and the resulting reaction of attachment/aversion trigger mental events that are, in turn, again sensations appraised as pleasant or unpleasant, which again result in reactions of attachment/aversion, and so on. It is a vicious cycle that is typically only interrupted by incoming stimuli that have a higher inherent priority than the ongoing mental activity and that often start the next chain of mental activity. Some interviewees reported that they escape this vicious cycle by observing the emotion without judgment (sometimes supported by analyzing how it feels, where it is felt, and if it changes over time), followed by a deliberate attempt to let the emotion go. The second step is accomplished by directing all attention to a sensation of the present moment (e.g., the sensations evoked by the breath), so that no attention is left for continuing the mental process. As, consequently, the mental process is irretrievably interrupted for that moment, the emotion vanishes. A sensation of the present moment might actually be the observed emotion itself. When the practitioner fully attends to the sensations connected with that emotion, no capacity is left for further processing the stimulus that triggered that emotion. Additionally, with this technique of observing the experiences of the present moment with full attention, the formation of emotions can be prevented to some extent. Some interviewees reported that during observation mode, they had no capacity left for the processing of additional thoughts (that would have referred to, for instance, the past, the future, fantasies, or interpretations). Thoughts are an important trigger of emotions and with their number and intensity reduced, emotional activity decreases.

Third, as described in Step 2 of the process model, practitioners try to “let go” expectations, convictions, esperances, and other cognitive concepts that have been built in the past and are stored in long-term memory. Letting go refers to the intentional deactivation of those concepts that were activated by a stimulus set in a certain situation. Since those concepts are crucial for the evaluation of a situation and the evaluation of the situation is crucial for the development of an emotion (see Lazarus and Folkman, 1984), following the deactivation, the momentarily arising or already existing emotion is terminated.

Fourth, by establishing an “observation mode,” meta-cognitive awareness is improved (see above). These improvements in meta-cognitive awareness lead to a faster realization that an emotion is coming up, and hence, emotion regulation strategies can be applied earlier and more effectively.

Finally, there is another mechanism that prevents emotions from arising and thus fosters equanimity. This mechanism is experienced in later stages in a meditation career since it requires a certain amount of deep meditative insights. These deep insights refer to the conditioned, transient and unsatisfactory nature of all phenomena (e.g., physical objects as well as mental states). First, these insights alter the cognitive representation of the characteristics of the own self/person (i.e., self-concept). The practitioner stops identifying with certain persons, objects, or ideas, and related convictions vanish. Second, even the experience of the own self in daily life (i.e., self-perception) alters in that it becomes less firm and solid. The practitioner feels that the self is conditioned and constantly changing. In the most extreme case, the perception of a self that is separate from the world vanishes. In the PROMISE model, these two phenomena (alteration of self-concept and self-perception) are termed “non-attachment.” In consequence of an advancing non-attachment, stimuli are appraised as threatening or desirable to a lesser extent since this judgment always refers to the consequences of the existence of that stimulus for the self: negative emotions often function as preservation from physical or psychological harm from the self. In particular, those emotions that are intended to maintain or protect the self-concept should sooner or later disappear completely in very experienced meditators.

##### Imitation

A last mechanism of Buddhist meditation practice is imitation or something one could term “from doing to being.” By means of a deliberate and effortful imitation of an intended mental quality, the habitual effortless dwelling in this quality is eventually achieved. That is, in the beginning of mediation practice, such a state does not arise automatically, but is intended for by particular behaviors. There are at least three such imitation processes. First, the active letting-go of cognitive concepts may be considered an imitation of the non-attachment to these very concepts. The difference is that before letting go, the concept is activated and gets potentially relevant for eliciting emotions that will be identified in a deliberate process before it is then deactivated. Non-attachment means that the concept possibly exists and gets activated in a certain situation, but since the concept is not connected to the self-concept, it does not elicit or maintain emotions. Second, the deliberate alteration of the affective quality of an experience imitates the quality of equanimity in a specific situation and in an actively regulating manner. Instead, equanimity itself is universal for all situations and it is automatized (or better de-automatized, because the automatic emotion activation does not happen anymore). The first and second imitation processes considered jointly, one can see that letting go of cognitive concepts in a certain situation leads to an alteration of the affective experience of this situation while non-attachment to cognitive concepts in general leads to an overall reduced emotional activity (i.e., equanimity). Third, the deliberately controlled observation mode parallels the effortless state of clarity freed from automatic thinking. The observation mode also parallels the state of equanimity.

In sum, the PROMISE model highlights equanimity (reduction in emotional reactivity) and insight (alteration of cognitions) as the two key effects of mindfulness meditation. These are the two effects that are actually intended by meditators and lead to positive long-term effects such as more wholesome mind states, improved social relationships, and fundamental alterations of self-concept and self-perception. The model comprehensively integrates the unique experiences of expert meditators, the contemporary Western concepts of mindfulness, and the ancient Buddhist concepts of mindfulness.

## Study 2: Examination of the Promise Model – a Quantitative Pilot Study

The PROMISE model derived in Study 1 is an empirically driven model based on qualitative data from interviews with meditation practitioners, refined by incorporating basic concepts from both ancient Buddhist scriptures and contemporary Western theorizing and research. As such, the model is intended to be comprehensive for what is nowadays termed “mindfulness meditation” and a sound basis for further research on both mindfulness and meditation. Yet, to propose the model as a reasonable foundation of further research, it needs to be tested with quantitative data. In a first attempt to do so, we set out to examine at least one manifestation of every step in the meditation process. Specifically, we analyzed if meditation practice (Steps 1 and 2) is related to the ability to observe individual experiences in a decentered way and to face experiences with openness and acceptance (Step 3), to the less frequent experience of self-related emotions and the more frequent experience of meditative insights (Step 4) as well as to an improved sense of satisfaction with life (Step 5). We also analyzed if both the meditation practice *per se* and the amount of meditation practice were able to predict the effects derived from the model. Additionally, we analyzed if the proposed relationships between the single steps in our model (see Figure 1) could be confirmed by the empirical data.

### Methods

#### Sample

Invitations for participation in an online survey were sent to meditation centers in Germany, Austria and Switzerland (mainly Rinzai Zen, Soto Zen, Tibetan Buddhism, Vipassana). To build a comparable control group, we contacted leisure-time groups that were concerned with technics, medicine, music, sports, art, social engagement, animal welfare, gardening, mental practices, and esoteric. In total, we obtained 484 complete data sets. We excluded participants who subjectively reported that they suffered from symptoms of a mental disorder at the time of measurement (such as depression, anxiety, eating disorders, stress disorder, etc.) and also participants who meditated according to a non-Buddhist tradition or had stopped their meditation practice at some time in the past.

There were 102 persons that meditated according to a Buddhist tradition (mean age: 48 years, *SD* = 12.7; 55% female; 51% with university degree; 94% German native speakers). According to their stated meditation tradition or teacher, they were classified into Theravada/Vipassana (*n* = 16), Vajrajana/Tibetan Buddhism (*n* = 34) and Zen Buddhism (n = 31) or miscellaneous Buddhist traditions (*n* = 21). Their mean meditation experience was 12 years (*SD* = 9.8 years).

The control group comprised 109 meditation-naïve persons and further 43 persons who had tried meditation at most a few times, resulting in 152 respondents (mean age: 40, *SD* = 16.11; 65% female; 39 with university degree, 99% German native speakers).

#### Material

##### Meditation practice

Participants answered if they were experienced in meditation (yes; yes, but I just tried; no) and only if they answered with „ “yes,“” i.e., they answered a comprehensive questionnaire including when they had started to meditate, when they had started to meditate on a regular basis, to which traditions their actual and former meditation techniques belong, how often they practiced per week, how long a single meditation session lasts, how many retreats they had attended, and when they had stopped their regular meditation practice (if applicable).

##### Observation mode

To assess the extent to which participants observe their experiences without being drawn into them, we used two subscales of a pre-version of the Fundamental Dimensions of Mindfulness Questionnaire (FuDiM; Eberth, 2016) with 4 items each: the subscale Undivided Attention to the Present Moment and the subscale Decentering/Metacognitive Awareness (that means the perception of thoughts and feelings without being absorbed by these experiences). Participants answered on a 6 point Likert scale from 0 = “I do not agree” to 5 = “I agree.” Internal consistency was medium to high in our samples: α_BudMed_ = 0.76, α_Control_ = 0.78 for Undivided Attention to the Present Moment and α_BudMed_ = 0.83, α_Control_ = 0.64 for Decentering/Metacognitive Awareness).

##### Deactivation of concepts stored in LTM

Openness/Acceptance was measured with 10 items that have been formulated on the basis of the interviews (Study 1). Examples are “I try to be rather open for my experiences instead of controlling or changing them” or “I try to accept every experience as it unfolds – whether it is pleasant or unpleasant”. Participants answered on a 6 point Likert scale from 0 = “I do not agree” to 5 = “I agree.” Internal consistency was high (α_BudMed_ = 0.92, α_Control_ = 0.90). Exploratory principal component analysis (PCA) revealed a one-dimensional solution (accounting for 56% of the variance) indicating that the items used represent a single construct.

##### Equanimity

To measure the frequency of intense emotions – a core feature of equanimity (see Study 1) – 108 students of the local university rated the intensity of 30 words that represent different emotional states in a pre-study. Eleven emotions were selected for assessment (concerned, irritated, disappointed, sad, frustrated, angry, impatient, desperate, resentful, yearning, proud). Participants answered how frequently they experienced these 11 emotional states in the last 6 months on a 6-point Likert scale ranging from 0 = “almost never” to 5 = “frequently.” Thus, a lower total score represents a higher level of equanimity. Internal consistency was high (α_BudMed_ = 0.85, α_Control_ = 0.80). Exploratory PCA revealed a one-dimensional solution (accounting for 33% of the variance).

##### Insight

During the qualitative interviews, various kinds of insights had been described. We clustered them semantically, resulting in the following categorization: insight in self-made suffering, insight in the subjectivity and relativity of own beliefs, insight in impermanence, insight in the connectedness of everything, and insight in the existence of inextricably contradictions. Twenty one items were derived from interview statements about the contents of meditative insights. Internal consistency was satisfactory (α_BudMed_ = 0.70, α_Control_ = 0.70). Participants answered on a 6 point Likert scale from 0 = “I do not agree” to 5 = “I agree.” Exploratory PCA revealed no dimensional structure in the data (i.e., the scree plot showed a smooth curve without any salient point), indicating that the items used do not correlate very strongly but also do not show any latent structure.

##### Life Satisfaction

The utilized Satisfaction with Life Scale (SWLS; Diener et al., 1985) consists of 5 items and quantifies how satisfied the respondents are with their lives. Internal consistency was medium to high in our samples (α_BudMed_ = 0.76, α_Control_ = 0.83).

### Results

**FIGURE 2:**
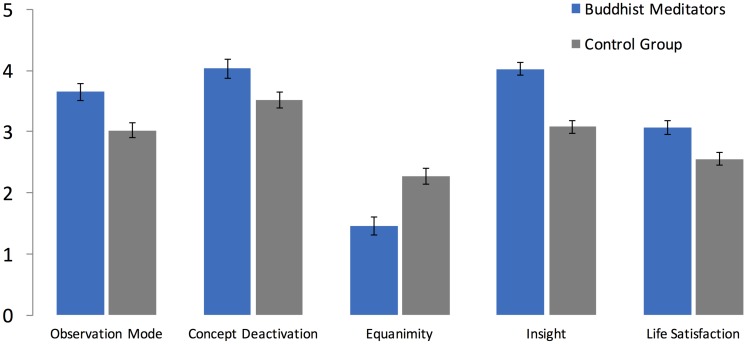
Means and 95%-CIs of five outcome variables for Buddhist meditators (*N* = 102) compared to a non-meditating control group (*N* = 152). Because equanimity is measured by the intensity of emotions lower values represent higher equanimity.

The *PROMISE* model proposes that the steady observing of mental experiences without being caught into them and the voluntary deactivation of concepts stored in long-term memory are mental actions that are executed both during and between meditation sessions. The abilities underlying these actions are trained when mindfulness practiced on a regular basis and, as a result, the mental actions can be established faster as well as executed longer and with less effort. By means of various processes (see above), habitual emotional activity is reduced and meditative insights are more likely to occur. As one of several consequences, mindfulness practitioners should become more satisfied with their life. We tested these hypotheses by comparing Buddhist meditators to a non-meditating control group on five variables: observation mode, concept deactivation, equanimity, insight and life satisfaction. As Figure 2 shows, Buddhist meditators report significantly higher values on all outcome variables (see Table 3 for exact values, effect sizes, and test statistics; see Supplementary  Material).

**TABLE 3:** Means, standard deviations and *t*-test statistics for the comparison between Buddhist meditators (*N* = 102) and the non-meditating control group (*N* = 152).

	**Buddhist meditators**	**Control group**			
	**Mean (*SD*)**	**Mean (*SD*)**	***d***	***t***	***P***
Observation mode	3.66 (0.70)	3.02 (0.77)	0.86	6.71	<0.001
Concept deactivation	4.04 (0.78)	3.52 (0.81)	0.65	5.10	<0.001
Equanimity	1.45 (0.77)	2.27 (0.81)	1.03	8.04	<0.001
Insight	4.02 (0.54)	3.08 (0.64)	1.56	12.10	<0.001
Life satisfaction	3.07 (0.54)	2.55 (0.66)	0.85	6.61	<0.001

*Because equanimity is measured by the intensity of emotions lower values represent higher equanimity.*

Are these differences between meditators and non-meditators a result of the presence vs. absence of meditation practice? Proper experiments to answer this question are difficult to run because prospective studies would have to span ten years or more to obtain measurable effects for some variables (e.g., meditative insights). Nevertheless, we can consider the characteristics of the meditation experience to get a tentative impression if formal practice has an influence on the outcome variables. This analysis will be described next.

#### Note on Analysis Strategy

We examined four aspects of individual meditation behavior (see Table 4). First, we obtained the *meditation experience* that spans from the beginning of the regular formal practice until time of measurement. It ranged from only a few days to 44 years in total (non-meditators excluded). Since the meditation experience necessarily correlates with participants’ age (*r* = 0.51) we used the age-corrected values when the outcome variable was also correlated with age. Second, we assessed the *mean duration* of single meditation sessions. It ranged from 12 to 120 min (*M* = 42 min, *Med* = 30 min, *SD* = 23.6). Third, we considered the *frequency* of formal meditation practice. The majority of participants meditated once (49%) or several times a day (32.4%). Some participants meditated several times a week but not daily (16.7%) and only 2% meditated only once a week. Fourth, we assessed the *number of retreats* attended by every meditator that ranged from none to “uncountable” retreats attended (*M* = 42 retreats, *Med* = 20 retreats, modus = 50 retreats, *SD* = 73.7).

**TABLE 4:** Influence of meditation experience characteristics on the outcome variables.

	**Meditation experience**	**Duration of single meditation sessions**	**Frequency of formal practice**	**Number of retreats attended**
**Observation mode**				
- Undivided attention to the present moment	*r*_corr_ = 0.26	*r* = 0.17	η^2^ = 0.06	*r* = 0.21
- Decentering/Metacognitive awareness	*r*_corr_ = 0.28	*r* = 0.26	η^2^ = 0.11	*r* = 0.37
**Concept deactivation**	*r* = 0.23	*r* = 0.06	η^2^ = 0.02	*r* = 0.25
**Meditative insight**	*r*_corr_ = 0.12	*r* = 0.19	η^2^ = 0.01	*r* = 0.28
**Equanimity**	*r* = 0.24	*r* = 0.01	η*^2^* = 0.07	*r* = 0.30

**r_*corr*_ is corrected for the influence of participants’ age on the observed variable, based on the data from the non-meditating control group. Concept deactivation and equanimity were not correlated with age.**

#### Observation Mode

Meditation experience correlated with both facets of the observation mode (*r*_undivided attention_ = 0.26, *p* = 0.008; *r*_decentering_ = 0.28, *p* = 0.005). The association with undivided attention was nearly linear, increasing slightly more strongly after 20 years of experience, while the association with decentering increases most in the first 15 years of formal practice. Furthermore, while for an increase of decentering it is most important to meditate at all, to develop a steady undivided attention, only repeated practice per day is significantly effective. Regarding the attendance of meditation retreats, the picture is similar: while for the development of an undivided attention, only a regular participation leads to strong effects, metacognitive awareness seems to develop rather continuously: the more retreats the stronger the effect (*r* = 0.37, *p* < 0.001).

#### Deactivation of Concepts Stored in LTM

Openness/acceptance correlated with meditation experience (*r* = 0.23, *p* = 0.02) but not with the duration of single meditation sessions (*r* = 0.06) nor the frequency of formal practice (η^2^ = 0.02). However, the attendance of retreats exerted a positive effect (*r* = 0.25, *p* = 0.01).

#### Insight

Insight did not correlate significantly neither with meditation experience (*r* = 0.12, *p* = 0.24) nor with meditation frequency (η^2^ = 0.01, *p* = 0.87), but to some extent with the duration of single meditation sessions (*r* = 0.19, *p* = 0.05). Regarding the number of retreats, the attendance of at least some retreats is more important than the total number of retreats. Only the very regular participation at such meditation events has a meaningful additional effect.

#### Equanimity

Equanimity correlated with meditation experience (*r* = 0.24, *p* = 0.01). How long single meditation sessions last does not seem to make a difference (*r* = 0.01, *p* = 0.91), but the frequency of formal practice has a certain influence (η^2^ = 0.07, *p* = 0.09). The attendance of meditation retreats seems to be particularly effective in reducing unwholesome emotional activity (*r* = 0.30, *p* = 0.003).

#### Path Model

**FIGURE 3:**
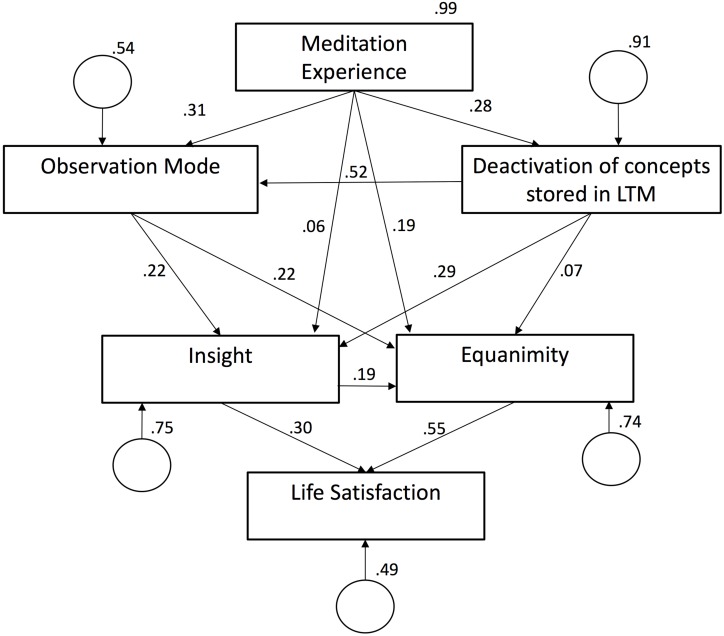
Standardized path coefficients (and *p*-values) for the proposed model. χ^2^ = 0.819 (*df* = 3, *p* = 0.845), *GFI* = 1.00, *NFI* = 1.00, *CMIN/DF* = 0.27, *RMSEA* < 0.001, *p-close* = 0.89; *N* = 102.

The relationships between the elements of the PROMISE model were tested using a path model (see Figure 3). To get a single score for meditation experience, we used factor scores composed from meditation experience, the number of retreats attended, and the meditation frequency. The model exhibits a very good fit with the data: χ^2^ = 0.819 (*df* = 3, *p* = 0.845), *GFI* = 1.00, *NFI* = 1.00, *CMIN/DF* = 0.27, *RMSEA* < 0.001, *p-close* = 0.89. The relationships proposed in our model were largely confirmed by the magnitude of the path coefficients: meditation practice leads to establishing and improving an observation mode (β = 0.31, *p* < 0.001) as well as to an attitude that increases the probability of deactivating concepts stored in memory (β = 0.28, *p* = 0.002). This attitude, in turn, promotes observing own experiences in the present moment (β = 0.52, *p* < 0.001). Hence, all of the hypotheses that were derived from Steps 2 and 3 of the PROMISE model were confirmed by the data. In Step 4, we postulated that the improvement of the trained abilities, the deliberate use of meditative mental actions in daily life, and the aspects that often accompany formal meditation practice trigger certain mechanisms that bring forth meditative insights and equanimity. These associations are represented in the path model by three arrows pointing to meditative insight and equanimity, respectively: deactivation of concepts stored in LTM leads to insights (β = 0.30, *p* < 0.001), the observation mode leads to meditative insights (β = 0.22, *p* = 0.035) and equanimity (β = 0.22, *p* = 0.035). Additionally, there is a direct effect of formal meditation practice on equanimity (β = 0.19, *p* = 0.025). And also, meditative insight fosters equanimity (β = 0.19, *p* = 0.025). Only small coefficients were found for the proposed associations between the deactivation of concepts stored in long term memory and equanimity (β = 0.07, *p* = 0.27) and the direct effect of meditation experience on meditative insight (β = 0.06, *p* = 0.27). As hypothesized in Step 5 of the PROMISE model, both meditative insight (β = 0.30, *p* < 0.001) and equanimity (β = 0.55, *p* < 0.001) heighten life satisfaction.

## Discussion

The PROMISE model postulates that mindfulness meditation leads (among other effects) to increased wellbeing (see also Lutz et al., 2008; Hölzel et al., 2011), mediated mainly by the establishment and progressive improvement of a mental mode that is characterized by observing all experiences of the present moment without being entangled with them, by the establishment and improvement of an attitude that fosters the deactivation of certain concepts stored in long-term memory, by meditative insights, and by equanimity. We presented a cross-sectional study with 102 meditating and 152 non-meditating participants and found higher scores for meditation practitioners in all outcome variables. Furthermore, we found that more meditation experience and a higher meditation intensity are associated with larger effects. Finally, we empirically tested the postulated associations between the variables in the PROMISE model, with data largely confirming the model. We shall now discuss the main findings and implications in detail.

### Observation Mode and Deactivation of Mental Concepts

Data confirmed our conjecture that mindfulness meditators score higher than non-meditators in observation mode and deactivating habitual concepts as they unfold. In addition, more meditation practice leads to higher scores within the meditating subsample. Still, there are some peculiarities. We divided the observation mode into two sub-concepts: Keeping undivided attention to the experiences of the present moment and observing own experiences without being entangled with them. While undivided attention seems to profit most from a tightly clocked regularity of sessions, decentering seems to improve continuously, with more experience and more practice leading to stronger effects. The voluntary deactivation of certain concepts stored in long-term memory is not affected by the practice schedule but instead by features of the meditation practice that represent a prolonged engagement with formal meditation or its context. Indeed, it seems likely that the actual deactivation in a particular situation is dependent on the ability and the volition or remembrance to deactivate or let go an automatically activated concept with all its associations. To remember this principle on a steady basis, it must be stored as an intrinsic goal, and to develop this goal, one has to understand why it is important. Reading the literature, listening to lectures of meditation teachers, and discussing ideas and questions with peers can foster this understanding. Thus, the effects of experience with meditation and retreats possibly reflect to some degree an intellectual engagement leading to a higher motivation to deactivate automatically activated concepts, with the eventual desire to make experiences that are as “pure” as possible.

### Insights

Meditative insights do not or only weakly correlate with the aspects of meditation intensity, with the exception of number of retreats. Notably, the effect of retreats is not gradual but more abrupt: It is very likely for a meditator to gain specific insights when attending the first retreats while the number of subsequent retreats does not seem to have a strong influence on the amount of insight. Some important features of most meditation retreats might help explain this effect. First, as already discussed above, retreats contain teaching lessons. The intellectual engagement with unknown or until then poorly understood ideas can lead to insights. Old scriptures, for instance, report that some of the Buddha‘s first disciples gained enlightenment by only listening to his teachings. Second, during retreats, practitioners meditate for up to 11 h a day (sometimes even longer). This can lead to deep absorption states and intense meditation experiences that the practitioner never experienced before. In accordance with the PROMISE model, those utterly novel experiences lead to deep meditative insights. Also, the duration of regular meditation sessions is associated with the attainment of meditative insights. Although for a moderate number of retreats, there is no correlation between this number and the amount of insight, this picture is different for large numbers of retreats: Participants who reported more than 60 retreats also reported significantly higher levels of insight. Of course, from our data we do not know whether more retreats lead to more insights or vice versa. We believe that the latter – more/deeper insights lead to more retreat visits – is the more likely direction of causality because practitioners who have gained very profound insights are very likely to feel a much stronger need for quiescence and withdrawal.

Not least, our path model contains a direct path from meditation experience to insights because we believed that states of absorption and deep meditative contemplation might have a direct effect on insight. There was, however, only a weak effect. Yet, we think that the absence of a stronger effect was probably due to methodological issues. First, the variable “meditation experience” was based on the meditation experience (in years) and meditation frequency but not on the duration of meditation sessions. Duration of meditation sessions raises the probability for deep concentrative states and correlated with the attainment of insights. Second, the instrument for assessing meditative insights is preliminary and in need for improvement that should include an extensive exploration of the content and the features of meditative insights, followed by the construction of a reliable measuring instrument. By now, there is only one such instrument (Ireland, 2013) that, however, captures insights in a rather rough and subjective way. Moreover, it can be applied only to (experienced) meditators and therefore, comparisons with non-meditators are impossible.

### Equanimity

Meditation experience has an influence on the emotional activity of a practitioner, as has the frequency of formal meditation in daily life and the number of retreats attended. However, the duration of regular meditation sessions has no influence. This might point to the fact that for the development of equanimity, processes are involved that are characterized by frequent repetition but that rely on dissipating resources so that a high number of repetitions in one block is not beneficial. It could also mean that equanimity mainly develops on the basis of processes that rely on a steady remembrance (as we discussed already with the deactivation of concepts). In the path model, the association between concept deactivation and equanimity was not strongly supported by our empirical data. One explanation might be that with the weakening resistance against negative emotions through the openness and acceptance associated with concept deactivation, those unpleasant experiences are felt more intensely and, therefore, emotions that had remained unconscious beforehand (emotional impulses that did not reach the threshold of consciousness) were now experienced consciously. In consequence, the total number of denoted emotions could remain the same, while the number of emotions affecting thoughts and behavior has decreased. A detailed study to examine the association between concept deactivation and equanimity would be commended. For this purpose, an alternative assessment for equanimity could be considered or constructed (for some first suggestions for the operationalization of equanimity, see Desbordes et al., 2014).

### Wellbeing

Buddhist meditators report greater levels of life satisfaction than persons that never meditated on a regularly basis. In our path model, we could show that the extent to which practitioners are satisfied with their lives is dependent on their insight and equanimity scores. Life satisfaction is the cognitive component of the wellbeing concept. Further research should investigate if insight and equanimity also influence other components of wellbeing, as well as other manifestations of effects defined by the PROMISE model that have not been investigated in this first study.

## General Discussion

The last decades of meditation research have brought about a large number of empirical findings. Now, it is time for integration and theorizing, in order to allow for a more exact evaluation of mindfulness meditation regarding its effectiveness for prevention, therapy, and wellbeing. Moreover, by knowing the precise processes of how meditation works, it becomes possible to evaluate interventions that are based upon mindfulness meditation regarding possible side effects, harms, and contra indications. Not least, ascribing the processes to specified effective components is necessary to improve existing interventions regarding effectivity and efficiency or to develop new interventions. At the moment, there is a great effort to build such theoretical models from which new and precise hypotheses can be derived. With the PROMISE model, we propose an approach that is driven by theory and empirical evidence (i.e., qualitative interviews with meditators on varying experience levels, ancient Buddhist scriptures, recent Western theorizing).

The most important characteristics of the PROMISE model include the differentiation of several steps in the whole process of meditation-induced transformation and the compilation of several mechanisms that work during the transformative process. Compared to other comprehensive exploratory approaches to meditation (e.g., Lutz et al., 2008; Hölzel et al., 2011; Dahl et al., 2015), the PROMISE model was constructed predominantly on the basis of in-depth interviews with meditators. Therefore, both novices and advanced meditators should find themselves in the descriptions of the relevant concepts and developmental process. The distinction between behavioral components, mental states during mindfulness practice, trained abilities, the two main effects *meditative insight* and *equanimity*, and distal effects like *wellbeing* or *interpersonal improvements* makes precise predictions about the sequence in which different effects unfold. The identification of behavioral components that might be connected to a formal meditation practice allows their isolated investigation and, therefore, the determination of variance explained by those components. Furthermore, by the explication of behaviors that are usually conducted alongside a formal meditation practice, it can be investigated if those behaviors represent necessary or even sufficient conditions for the occurrence of certain effects and processes. We conducted a first examination of the PROMISE model with a cross-sectional questionnaire study. Of course, this design underlies shortcomings such as no evaluation of the direction of causes and effects, self-selection of participants, or response tendencies. It would be desirable to test single predictions of the model with more rigorous experimental designs.

The main building blocks of the PROMISE model are two novel effects of mindfulness meditation practice, namely the attainment of *equanimity* and meditative *insights*. Despite their central role in our model (and even in the Buddhist Eightfold Noble Path), prior research has widely neglected these two effects. One reason for this omission might be that these effects might occur relatively late in a meditation career or – with regard to meditative insights – are rare. Therefore, they are not well suited for rigorously controlled experimental designs. Another reason could be that, to date, these two important effects are lacking coherent conceptualization and measurement. Our definition of equanimity is mainly informed by a Western understanding (see, e.g., Desbordes et al., 2014, for an alternative definition), and more discourse among scientists and practitioners is needed to arrive at a consensual definition. The same is true for meditative insights. There are few approaches to defining and/or operationalizing meditative insights (Grabovac et al., 2011; Ireland, 2013). Further research is needed, especially regarding the concrete content and characteristics of meditative insights as well as attempts to separate them from insights that are attained in daily life or psychotherapy, for instance. Having said this, it is also important to note that for three variables – namely deactivation of concepts stored in LTM, equanimity, and insight –we had developed questionnaires based on the qualitative data from Study 1. Although all these scales showed sufficient internal consistency and no latent dimensional structures, they must be treated with caution. To analyze the validity of these scales by additionally comparing them with existing questionnaires measuring mindfulness or related concepts would have been far beyond the scope of the present study, most importantly because it is a delicate endeavor to keep experienced meditators in line when doing empirical studies. Thus, future research should focus on the advancement and validation of appropriate measurement instruments.

Regarding both equanimity and meditative insights, we need a thorough discussion about how to measure them (be it qualitatively or quantitatively). It is, for instance, likely that equanimity develops in concrete consecutive steps. Hints for such a discontinuous development can be found in the old Buddhist scriptures (e.g., in the Satipatthana Sutta I 55-63) that distinguish worldly and spiritual feelings and propose that the latter cannot be overcome until the former are successfully abandoned. The development of insight may also occur in discrete steps that could be consecutive or independent of each other. Either way, we should intensify the investigation of the nature of the insights. In our qualitative study, experiences with insights were very diverse. Future studies will have to identify whether there are common patterns of insights and whether there are specific sequences in which these insights unfold in the course of meditative practice.

## Data Availability Statement

All quantitative datasets of Study 2 is included in the Supplementary Files. The qualitative data of Study 1 are available on request to the corresponding author.

## Ethics Statement

Ethical review and approval was not required for the study on human participants in accordance with the local legislation and institutional requirements. The participants provided their written informed consent to participate in this study.

## Author Contributions

JE, PS, and TS developed the data collection and analysis strategies. JE developed the theoretical framework of the article and wrote most parts of the manuscript. PS and TS completed and revised the manuscript. Coding and the development of categories and the final model was done by all authors.

## Conflict of Interest

The authors declare that the research was conducted in the absence of any commercial or financial relationships that could be construed as a potential conflict of interest.

## Footnotes

1 http://www.audiotranskription.de
